# Phylogenetic distribution of plant snoRNA families

**DOI:** 10.1186/s12864-016-3301-2

**Published:** 2016-11-24

**Authors:** Deblina Patra Bhattacharya, Sebastian Canzler, Stephanie Kehr, Jana Hertel, Ivo Grosse, Peter F. Stadler

**Affiliations:** 1 Bioinformatics Group, Dept. Computer Science, and artin-Luther-Universität Halle-Wittenberg, Leipzig, D-04107 Germany; 2 Institut für Informatik, Halle (Saale), D-06120 Germany; 3 Max Planck Institute for Mathematics in the Sciences, Inselstraße 22, Leipzig, D-04103 Germany; 4 Fraunhofer Institute for Cell Therapy and Immunology, Perlickstrasse 1, Leipzig, D-04103 Germany; 5 Department of Theoretical Chemistry of the University of Vienna, Währingerstrasse 17, Leipzig, A-1090 Germany; 6 Center for RNA in Technology and Health, Univ. Copenhagen, Grønnegårdsvej 3, Frederiksberg C, Copenhagen, Denmark; 7 Santa Fe Institute, 1399 Hyde Park Road, Santa Fe, NM 87501 USA; 8 German Centre for Integrative Biodiversity Research (iDiv), Halle-Jena-Leipzig, Leipzig, Germany; 9 Young Investigators Group Bioinformatics & Transcriptomics, Helmholtz Centre for Environmental Research – UFZ, Permoserstrasse 15, Leipzig, D-04318 Germany

**Keywords:** snoRNAs, Evolution, Small RNAs, snoRNA targets

## Abstract

**Background:**

Small nucleolar RNAs (snoRNAs) are one of the most ancient families amongst non-protein-coding RNAs. They are ubiquitous in Archaea and Eukarya but absent in bacteria. Their main function is to target chemical modifications of ribosomal RNAs. They fall into two classes, box C/D snoRNAs and box H/ACA snoRNAs, which are clearly distinguished by conserved sequence motifs and the type of chemical modification that they govern. Similarly to microRNAs, snoRNAs appear in distinct families of homologs that affect homologous targets. In animals, snoRNAs and their evolution have been studied in much detail. In plants, however, their evolution has attracted comparably little attention.

**Results:**

In order to chart the phylogenetic distribution of individual snoRNA families in plants, we applied a sophisticated approach for identifying homologs of known plant snoRNAs across the plant kingdom. In response to the relatively fast evolution of snoRNAs, information on conserved sequence boxes, target sequences, and secondary structure is combined to identify additional snoRNAs. We identified 296 families of snoRNAs in 24 species and traced their evolution throughout the plant kingdom. Many of the plant snoRNA families comprise paralogs. We also found that targets are well-conserved for most snoRNA families.

**Conclusions:**

The sequence conservation of snoRNAs is sufficient to establish homologies between phyla. The degree of this conservation tapers off, however, between land plants and algae. Plant snoRNAs are frequently organized in highly conserved spatial clusters. As a resource for further investigations we provide carefully curated and annotated alignments for each snoRNA family under investigation.

**Electronic supplementary material:**

The online version of this article (doi:10.1186/s12864-016-3301-2) contains supplementary material, which is available to authorized users.

## Background

Small nucleolar RNAs function as guides in site-specific RNA modification [1, 2]. They fall into two distinct classes: box H/ACA snoRNAs responsible for targeting pseudouridylation sites and box C/D sno-RNAs directing 2’-O-methylation of ribonucleotides. Both are part of well-defined ribonucleo-particles the snoRNPs [3]. SnoRNAs are evolutionarily ancient. Their origin pre-dates the divergence of Archaea and Eukarya [4] and thus also the origin of their namesake, the nucleolus. Mostly, snoRNAs target ribosomal RNAs. Subclasses of snoRNAs that usually localize to the Cajal bodies, often referred to as scaRNAs, are responsible for methylation and pseudouridylation in particular of spliceosomal snRNAs [5].

In vertebrates, mature snoRNAs are mainly produced from introns of precursors that can be both protein-coding mRNAs or non-coding “host genes.” In contrast, only a few snoRNAs are intronic in budding yeast and plants [6, 7]. Moreover, the loss of introns through widespread degeneration of splicing signals has lead to snoRNA host genes that carry snoRNAs as exons in yeast [8].

There is a tendency for polycistronic snoRNA precursors in general. In plants, however, polycistronic precursors are the standard [9–11]. Individual snoRNAs are usually excised from their precursor transcript by RNase III endonucleases and then trimmed by exonucleases [12, 13]. The ends of mature snoRNA are then protected from further degradation by the assembly of snoRNP core proteins [14]. A curious exception are the tRNA(Gly)-snoRNA and tRNA(Met)-snoRNA cotranscripts in dicots and monocots, respectively [15].

Box C/D snoRNAs share the conserved sequence motifs C (RUGAUGA) close to the 5’-end and D (CUGA) near the 3’-end, which are tethered by a terminal stem-loop. In addition, internal C’ and D’ box can be found in many of the box C/D snoRNA. These motifs have the same consensus sequence as the C and D boxes, resp., but show a higher level of variation in both animals and plants. The assembly of box C/D snoRNPs involves the formation of a kink-turn (K-turn) motif [16, 17]. This involves the the alignment of the C and D boxes and the formation of a crucial non-canonical G:A pair across the asymmetric bulge [18–21].

The box H/ACA snoRNAs are distinguished by the presence of an ACA triplet at their 3’-end and a characteristic hairpin-hinge-hairpin-tail secondary structure with the H box (ANANNA) located in the hinge region [22, 23].

The conserved sequence motifs (C, D’, C’, D, H, and ACA) serve as binding sites for protein components of the snoRNPs. Both classes of snoRNAs recognize their targets by complementary base pairing. The antisense elements of box C/D snoRNAs are located immediately upstream of the boxes D and D’ and have a typical length of 10-15nt. The antisense elements of box H/ACA snoRNAs are located within interior loops that interrupt the hairpins, see e.g. [2].

Beyond their function as guides for chemical modifications, a few snoRNAs are required for the cleavage of the ribosomal RNA precursors [24], among them in particular the U3 and the U14 snoRNAs. In contrast to the modification guides, these snoRNAs are essential for cell survival in human and yeast. They are also ubiquitously present throughout eukaryotes [25–27]. Some snoRNAs are involved in regulating gene expression, e.g. by modulating mRNA splicing or editing [2, 4]. More recently, snoRNAs have also been identified as a source of miRNA-like small RNAs that function in mRNA silencing found in diverse organisms from archaea to humans [28, 29]. SnoRNAs have even been found to be important players in cancer, suggesting that they fullfil multiple additional function in cellular regulation [21, 30].

Based on sequence similarity, snoRNAs fall into many well-defined families of homologous genes. As a consequence of the frequent segmental, chromosomal, and whole genome duplications in plant genome evolution, most plant snoRNA families have multiple paralogous members both in spatial clusters and spread throughout the genome [29].

Despite their ancient ancestry as a class [31], the long-term evolution, of individual snoRNA families across clade borders, has not been solved, comprehensively.

Several studies showed that many snoRNA families are conserved at phylum or even kingdom level in animals [32], plants [9], and fungi [33]. The genome-wide analysis of chicken snoRNAs provided direct evidence for extensive recombination and separation of guiding function [34]. Similarly, multicellular fungi exhibit a more complex pattern of methylation guided by box C/D snoRNAs than unicellular yeasts [35]. Nevertheless, conserved snoRNA targets typically have conserved modification sites, although there is some redundancy and an appreciable level of turnover throughout the animal kingdom [32].

Matching the situation in microRNAs [36], there is evidence for clade specific de-novo innovation of novel snoRNA families found in fungi, platypus as well as in humans [1, 37, 38]. The gist of the study is that so far there is no clear picture if and how the evolution of plant snoRNAs differs from the situation in fungi although a lot of data are available, dispersed throughout the literature.

A survey from 2010 concludes that we are still far from a comprehensive picture of snoRNA evolution and many more snoRNAs of both known and novel families remain to be found [39]. Recent experimental work has turned up many new snoRNA families even in the very well-studied genomes of human and fly [38, 40, 41].

Although there is good evidence for the conservation of many of the chemical modification sites on rRNAs and snRNAs between eukaryotic kingdoms [42], it is still an open question to what extent individual snoRNA families are homologous at such large phylogenetic distances. This is difficult to address since snoRNA sequences evolve quite rapidly apart from the conserved boxes and the antisense region. Only on the basis of detailed analysis of the conservation of snoRNA homologous within kingdoms it is possible to draw conclusions on the pattern of long-term evolution on snoRNA families also bridging clade and kingdom borders.

In this contribution we reconstruct the evolutionary history of snoRNAs in the plant kingdom. We focus on the identification of additional homologs in considered plant genomes and focuses on interesting patterns of conserved snoRNA families and regions of clustered snoRNAs. For each snoRNA family the evolution is systematically traced back to its last common ancestor.

## Results and discussion

From the intial set of collected and curated snoRNA families, snoRNAs are mapped to all the plant genomes and family-wide alignments of all retained candidate sequences were calculated. Finally, a putative history of gains and losses of genes within each snoRNA family was constructed. The initial query set of 554 snoRNA genes was comprised of a collation of all available (plant) snoRNA databases. These sequences were assigned to 222 box C/D and 74 box H/ACA snoRNA families after manual curation and annotation of the box C/D and box H/ACA snoRNAs. We identified a total of 5116 additional homologs in the 24 plant species under consideration.

### Heatmaps of snoRNA families

**Fig. 1:**
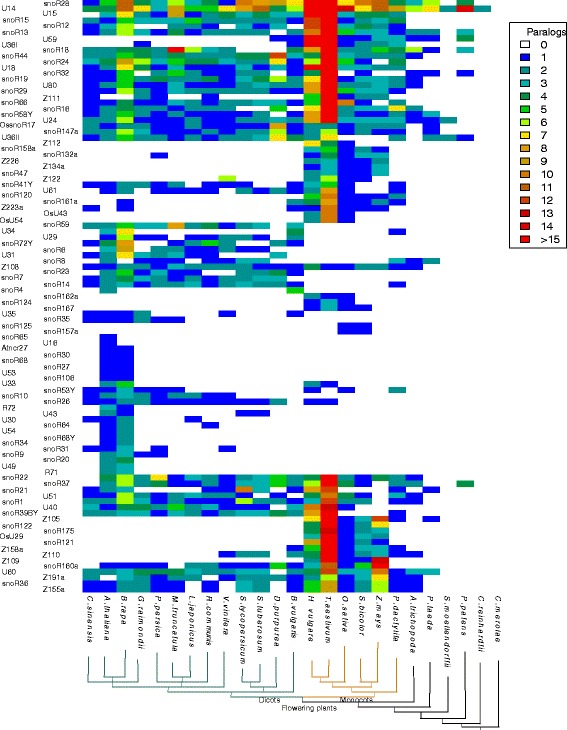
Heatmap of box C/D snoRNAs. The heatmap (built in R with heatmap.2 version) shows the box C/D snoRNA families and their distribution amongst the plant species. The colour code reflects the number of box C/D paralogs found within each species. The phylogenetic tree was constructed from recent literature and NCBI Taxonomy information

**Fig. 2:**
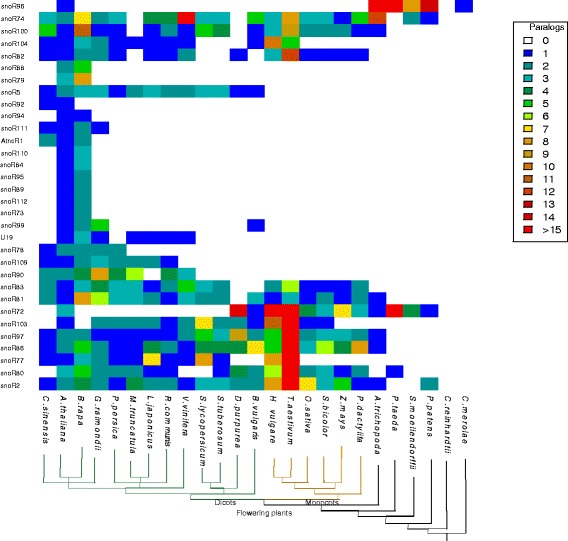
Heatmap of box H/ACA snoRNAs. The heatmap (built in R with heatmap.2 version) shows the box H/ACA snoRNA families and their distribution amongst the plant species. The colour code reflects the number of box H/ACA paralogs found within each species. The phylogenetic tree was constructed from recent literature and NCBI Taxonomy information

The phylogenetic distribution of the snoRNA families is shown in Figs. 1 and 2 in form of heatmaps color-coding the number of family members. The relevant csv files are provided as Additional files 1 and 2. SnoRNA families that are found only in one species such as in *Arabidopsis*, *rice*, or *Chlamydomonas* are not shown in the heatmaps. For the heatmaps only the 110 snoRNA families that were found to be conserved in more than one species are selected.

Several patterns are apparent. With the exception of the highly conserved U14 family and the snoR96 family that shows a much more scattered distribution, snoRNAs from land plants do not have identifyable homologs in green algae. Seven families of box C/D snoRNAs (snoR28, U14, snoR13, snoR18, snoR32, U36II, and snoR37) are conserved in land plants. Among these U14 is present nearly ubiquitously. Missing sequences in single species (white cells) are most likely caused by unidentifiable homology due to rapid snoRNA evolution rather than representing true snoRNA losses.

**Fig. 3:**
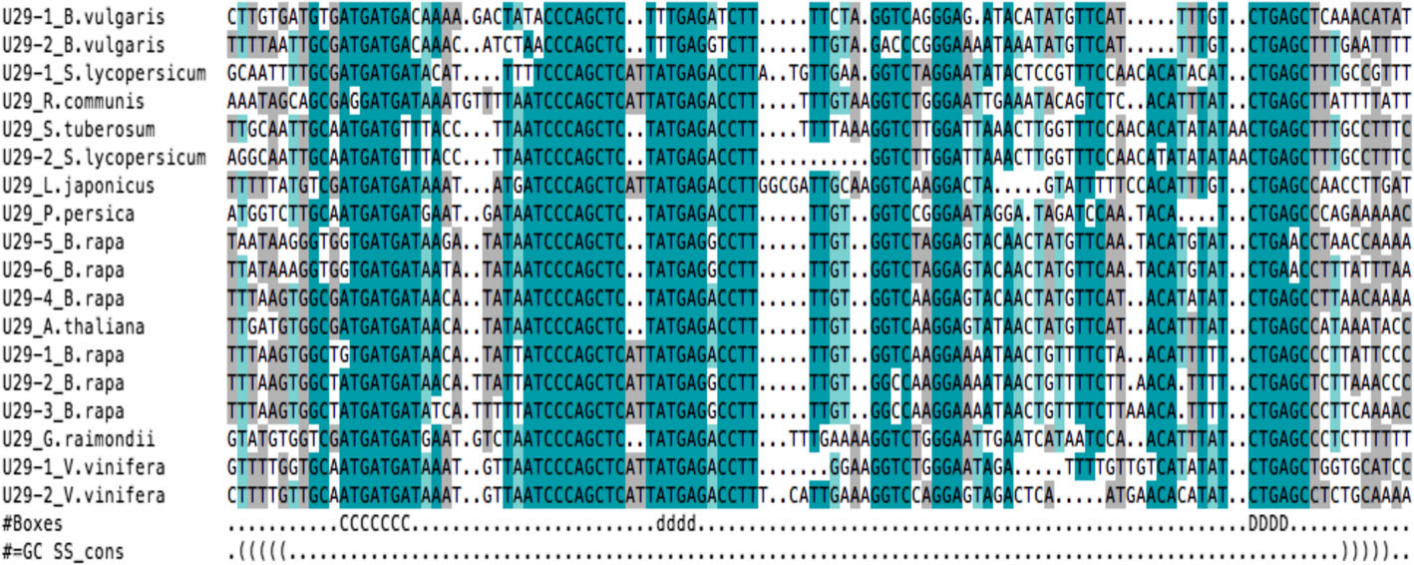
Conserved snoRNA box motifs. Conservation pattern of snoRNA U29. In the #Boxes line nt marked with C, D, and d belong to the box C, box D, and box D’, respectivley. The consensus secondary structure in dot-bracket notation provides the typical terminal stem with the unpaired nucleotides inbetween. The region upstream of the box D’ is highly conserved. It is the putative antisense element for guiding a modification. The region upstream of the box D is less conserved than box D’

Four H/ACA snoRNA families (snoR2, snoR72, snoR96, and snoR74) are present throughout the land plants, albeit only snoR2 was found in almost all species investigated here. The largest fraction of identified snoRNAs (76 box C/D and 20 box H/ACA families) are common to the flowering plants including both monocots and dicots. Target prediction employed by the snoStrip pipeline [33] suggests that 12 of the target sites in rRNAs are conserved throughout the plant kingdom (Additional file 3). It is possible that many of these families are in fact evolutionarily older and that the apparent restriction to land plants or flowering plants is a consequence of the limited sensitivity of state-of-the-art homology search methods. The consensus box motifs within some snoRNA families are very well conserved across the plant kingdom, see Fig. 3 for an example.

On the other hand, there are many families with a very narrow phylogenetic distribution: 27 families are found only in *Arabidopsis*, e.g. snoR107, 28 families appear to be specific to *Oryza*, e.g. snoR146a, and 131 families appear only in *Chlamydomonas*, e.g. CrACA02. Most of the *Arabidopsis*-specific snoRNAs have been reported to have their targets in ribosomal RNAs [43]. Either these sequences have evolved extremely rapidly, essentially at neutral rates, or they are true species or genus-specific innovations. The uneven distribution of snoRNAs across the investigated species most likely is an artefact: systematic experimental surveys for snoRNAs been conducted in particular for *Arabidopsis*, *Oryza*, and *Chlamydomonas*. For other species much less extensive data have been reported in the literature, hence most of the snoRNA genes are annotated by homology.

A very interesting pattern is the large block of box C/D snoRNAs (20 families) that is only present in monocots. A similar pattern is not visible for box H/ACA snoRNAs. There is also no such pattern of dicot-specific box C/D snoRNAs or dicot-specific box H/ACA snoRNAs. Hence, it is very unlikely that the monocot specific families of box C/D snoRNAs are just an artefact caused by limitations in the homology search method. So they should be interpreted as true monocot innovations.

Finally, focussing on column-wise patterns we observe a systematically elevated number of snoRNA paralogs in some species. Examples include *Brassica rapa* and *Digitalis purpurea* among dicots, as well as *Triticum aestivum* and *Hordeum vulgare* among monocots. By comparison with the Plant Genome Duplication Database [44] this observation is readily explained by phylogenetically recent genome duplication or triplication events.

There are several reasons why snoRNAs appear to be missing in some species or clades. First, we may see true gene losses. A second explanation is that they have diverged beyond our ability to detect and identify them by any of the available methods of homology search. This a likely explanation in particular for large phylogenetic distances. Third, incomplete genome assemblies can explain apparent gene losses. This explanation is plausible in particular for scattered, non-systematic “white spots” in the heatmaps.

### snoRNA clusters

SnoRNAs that are encoded or positioned closely together in the same chromosomal region are considered as “snoRNA clusters”. In order to study the long-term integrity of those clusters we investigated representative examples: the 68 rice snoRNA clusters described in [10]. Multiple snoRNA clusters have also been identified and studied in some detail in *A. thaliana* [45]. In this case, we find 10 snoRNA clusters that are conserved in rice and at least in some of the selected 24 plant species considered here, 5 of which have also been described in *A. thaliana* [45].

**Fig. 4:**
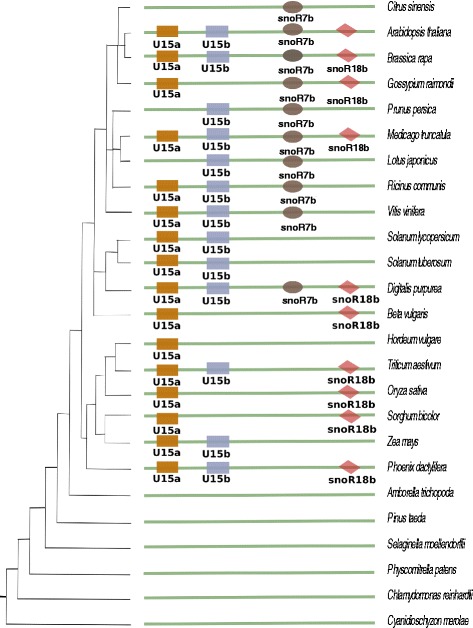
U15a-U15b-snoR7b-snoR18b cluster. Evolutionary observation of snoRNA “U15a-U15b-snoR7b-snoR18b cluster”, where we find two members of the U15 family (U15A and U15B) and snoR18b date back to the magnoliophyte ancestor (*P.dactylifera*), whereas snoR7b seems to be a recent innovation [10]

The 10 genomic clusters involve 22 distinct snoRNA families. A subset of the clusters comprises highly conserved snoRNAs, whereas most of the rice clusters are not conserved in other species. Several snoRNA families have members in distinct clusters. Figure 4 summarizes the evolutionary history of “U15a-U15b-snoR7b-snoR18b cluster” termed “cluster 5” in rice [10], which consists of U15a, U15b, snoR7b, and snoR18b, respectively. While two members of the U15 family (U15A and U15B) and snoR18b date back to the magnoliophyte ancestor (*P. dactylifera*), snoR7b is a more recent addition, incorporated in the dicot ancestor. Its homolog in *A. thaliana* was discussed in [45] as the “U15a-U15b-snoR7.1 cluster”.

Details on the 9 other conserved clusters (1, 19, 20, 43, 49, 53, 56, 58, and 66) in the terminology of [10]) are provided as Additional file 4. The U36Ia-U36IIa-U36IIb cluster named as “cluster 1” in rice is only present in the flowering plants. In the snoR12-U24 cluster (“cluster 19”), which was termed “U12.2-U24.2 cluster” in *A.thaliana* [45], U24 was present already in the ancestor of viridiplantae. In contrast, snoR12 originated later in the mesangiospermae or the flowering plants. In cluster snoR22a-snoR23-snoR22b (“cluster 20”), the *A. thaliana* “U32.2-U27.2-U80.2 cluster” [45], snoR22b dating back to the magniliophyte ancestor whereas, snoR22a appears in the monocots and also in few recent dicot plants. However, snoR23 is the prominent addition in the dicot plants. In cluster U27-U80b (“Cluster 43”), amongst U27 and U80b, U27 is the recent snoRNA appearing in the mesangiospermae family, while U80b can be traced back to magniliophyta. It is also found in *A. thaliana* [45] as the “U32.2-U27.2-U80.2 cluster”. In the cluster U61-snoR14 (“cluster 49”) corresponding to the “U61-U14.1-U56” cluster” in *A.thaliana* [45], both U61 and snoR14 appear in the measangiospermae family, however, snoR14 is more consistently conserved in the mesangiospermae plant species. Cluster snoR44-snoR17-snoR147a (“cluster 53”) consists of snoR44, snoR17, and snoR147. snoR147 is the ancestral snoRNA dating back to spermatophyte ancestor, followed by snoR44 dating back to the magniliophyte ancestor, whereas snoR17 appear to be recent emergence in the mesangiospermae or flowering plants. snoR167-snoR47 cluster (“cluster 56”) comprising snoR167 and snoR47, both of them appear only in the monocots without any innovation in the recent species. In cluster snoR53Y-U29a-U29b cluster (“cluster 58”), although snoR53Y emerges in the mesangiospermae family but is not consistently conserved throughout but also re-appears in recent dicots, whereas both U29a and U29b are restricted to monocots. Cluster U43a-snoR16 (“cluster 66”) comprising U43a and snoR16, snoR16 seems to date back to magnoliophyte ancestor whereas U43a although is a recent addition but restricted to subfamily BOP Clade. This cluster is also already mentioned in *A. thaliana* [45] as “snoR16.1-U43.1 cluster”. The conservation of many snoRNA clusters independently strongly supports the results of the homology-based family assignments.

### snoRNA targets

**Fig. 5:**
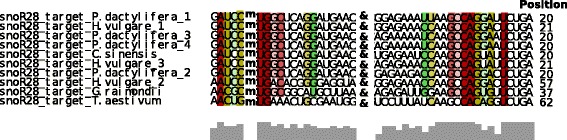
SnoRNA28 target conservation. Conservation of the interaction between the region upstream of D-box of snoRNA family snoR28 (*right side*) and the region around the 2’-O-methylated cytosine in 18S rRNA (*left side*). Target RNA segment and ASE are separated by &. The methylated residue is marked with M. The position of the predicted modification in the 18S rRNA sequence within each species is given at the end of each row. *Red* and *green* columns highlight conservation of the RNA-RNA interaction. Completely conserved base pairs are shown in *red*. *Green* columns mark base pairs with compensatory mutations. *Lighter colors* indicate loss of base pairs in individual species. The *gray* bars at the bottom correspond to the degree of sequence conservation. The last three snoR28 paralogs are more divergent and presumably address different targets

Systematic prediction of snoRNA targets in rRNAs and snRNAs showed that known and many predicted targets are usually conserved when the snoRNA is conserved. The complete archive of rRNAs and snRNAs used for target prediction is provided as Additional file 5. As an example, Fig. 5 shows the targets for snoR28 in the ribosomal RNA 18S as predicted by LocARNA [46]. While we were able to identify putative targets for most snoRNA families, several orphan snoRNAs (where no target RNAs are found) remain: snoR8, snoR9, snoR106, snoR107, snoR109, snoR112, CrCD72, CrCD74, CrACA54, and CrACA55. Orphan snoRNAs for which we could not find any rRNA or snRNA target may have a different function, e.g. they may target other RNAs such as mRNAs, or they may act as precursor molecules for the production of small regulatory RNAs [11].

### Evolution of snoRNA families

**Fig. 6:**
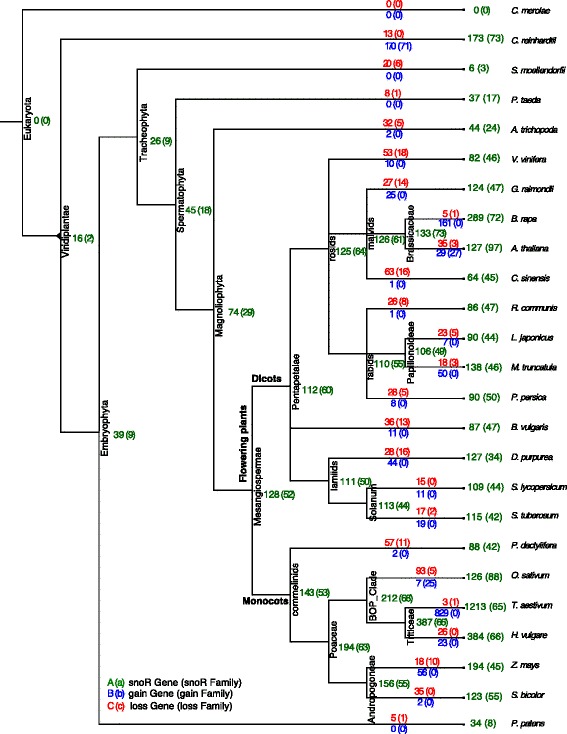
Phylogenetic tree of box C/D snoRNAs. Phylogenetic tree of C/D snoRNAs of 24 plant species and red alga (*C. merolae*). The phylogenetic tree was constructed from recent literature and NCBI Taxonomy information. The species are assigned to the leaves. ePoPE was applied to each snoRNA family individually (data not shown). To retrieve an impression about the evolution of all snoRNA families these individual results were summarized, again using ePoPE. The numbers are the results of this summary. Green numbers refer to the predicted number of observed genes (families) at each node. Red numbers refer to the number of lost genes (families) while blue numbers to the number of gained genes (families)

To draw a comprehensive picture of the snoRNA evolution in the 24 plant species we used the compational approach ePoPE [47]. It implements a parsimony-based presence/absence analysis of genes within a gene family. Given the phylogenetic tree of our plants of interest and the built alignments this program systematically traced each individual snoRNA family back to its last common ancestor. The ePoPE program also returns a most parsimonious solution for the history of gains and losses of genes along the phylogenetic tree. A summary of this study over *all* plant snoRNA families is given in Figs. 6 (box C/D snoRNAs) and 7 (box H/ACA snoRNAs). For each snoRNA family we provide the individual ePoPE results in machine-readable form, see Additional files 6 and 7. These include the annotation of (i) the last common ancestor of this snoRNA family, (ii) the predicted number of snoRNA genes that emerged and diverged at each branch and (iii) the number of genes that is observed in the species (at the leafs).

**Fig. 7:**
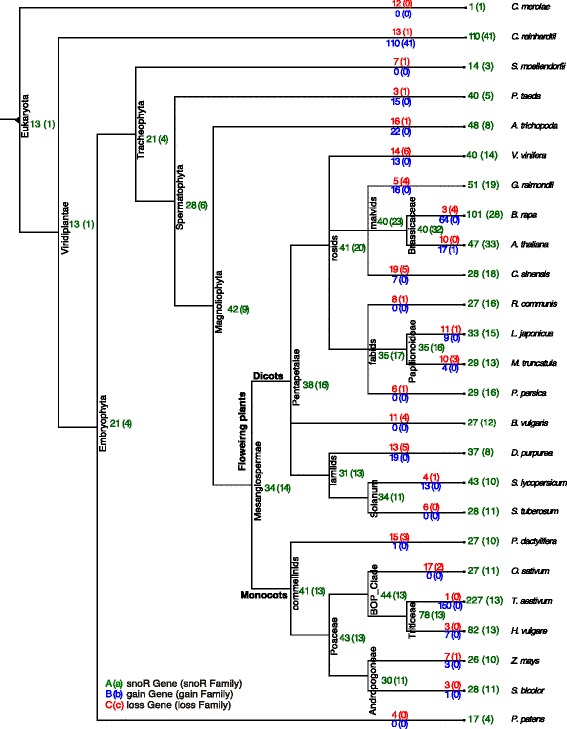
Phylogenetic tree of box H/ACA snoRNAs. Phylogenetic tree of H/ACA snoRNAs of 24 plant species and red alga (*C. merolae*). The species are assigned to the leaves. ePoPE was applied to each snoRNA family individually (data not shown). To retrieve an impression about the evolution of all snoRNA families these individual results were summarized, again using ePoPE. The numbers are the results of this summary. Green numbers refer to the predicted number of observed genes (families) at each node. Red numbers refer to the number of lost genes (families) while blue numbers to the number of gained genes (families)

## Conclusions

Many snoRNA families are deeply conserved in the plant kingdom. Surprisingly, only a few families can unambiguously be traced back to the ancestor of land plants. Some families are innovations that emerged later during plant evolution. We hypothesize that at least 8 snoRNA families are recent innovations, i.e. snoR59, U29, snoR72Y, snoR6, U31, snoR8, snoR23, and snoR7. This hypothesis is supported by a large group of monocot-specific snoRNAs. The strong conservation of some chemical modification sites in ribosomal RNAs, however, supports the idea that there is a core of snoRNA genes that are ubiquituously present in Eukarya and possibly even in Archaea. The small size, the relative fast rate of evolution, and limitations of available homology search techniques, however, make it hard to directly test this hypothesis. Surprisingly, homology search methods fail, with very few exceptions, to identify homologs of landplant snoRNAs in green algae. We suspect, however, that this rather a limitation of the state of the art in homology search.

Despite these and many other limitations, several interesting patterns on snoRNA evolution in plants can be observed. Many snoRNA families have well-identifiable paralogs. Furthermore, distinction between evolutionarily old families and a collection of evolutionarily young innovations is observed see Figs. 1 and 2. The latter requires a more detailed investigation of closely related species. The rapidly increasing collection of completely sequenced rosids, for example, may serve as an excellent starting point for a systematic study of snoRNA turnover.

The nomenclature of plant snoRNAs is often species specific and it respects only partially known orthology relationships at the level of individual snoRNAs families. In particular, this is the case where data go beyond the plant snoRNA database [48]. In some cases, such as the U29/U29a, U54/U54a, or snoR68Y/snoR68 (also named CrCD03), naming convention for different species are even contradictory. This poses a serious obstacle for large-scale comparative studies and causes the danger of mis-interpreting the results of comparative surveys. In this contribution, we used the *Arabidopsis* or *Oryza* names for snoRNA families wherever possible based on the assumption that these are most widely used. A comprehensive table of synonyms is provided as Additional file 8. A nomenclature of plant snoRNAs that, similar to the micro RNA nomenclature, is (a) designed to be applicable to all (land) plant species, (b) strives to honor homologies, and (c) distinguishes box H/ACA and box C/D snoRNAs would be highly desirable and would greatly facilitate comparative studies.

Here, we provide a comprehensive, well curated collection of homologous snoRNAs in 24 plant species evenly covering the plant kingdom. For each individual snoRNA family we prepared multiple sequence alignments in the Rfam-compatible STOCKHOLM^1^ format (see Additional file 9). Apart from the aligned sequences these files contain the predicted conserved secondary structure and the positions of the characteristic box motifs of snoRNAs. In addition, all data regarding target prediction, snoRNA distribution and evolution can be downloaded on the supplement page. These results might become a valuable resource for more detailed studies on snoRNAs and their evolution in the plant kingdom.

## Methods

### Data sources

We selected 24 plant species with completely sequenced genomes covering the plant kingdom, see Figs. 1 and 2. Among crown group (living representatives of the collection together with their ancestors back to their most recent common ancestor as well as all of that ancestor’s descendants) eudicots, we preferrentially included species for which snoRNAs had been described in the literature.

We collected all available plant snoRNA sequences from the SnoRNA orthologous gene database (SNOPY [43]) and the plant snoRNA database [48]. In addition we extracted snoRNA sequences from the literature [10, 45, 49–53].

We considered only the rRNAs/snRNAs as potential targets. Ribosomal RNA sequences of the 24 plant and red algae species are downloaded from the SILVA database [54]. The snRNAs comprising of U1, U2, U4, U4atac, U5, U6, U6atac, U11, and U12 are imported from datasets of the plantDARIO webserver [55].

### Curation of initial snoRNA data

From the initial set of collected snoRNAs, the box motifs are annotated and categorized into box C/D and box H/ACA snoRNAs. The characteristic boxes (C, D’, C’, D, H, ACA) are annotated manually using the sequence patterns as constraints given in [56].

Previous analyses from the Bachellerie laboratory showed conserved spacing between the box C/D core motif and the internal D’/C’ motif of the archaeal box C/D snoRNAs [57]. Although alteration of D and D’ spacer distances does not affect box C/D and D’/C’ RNP assembly, the spacer distances severely affect box C/D and D’/C’ RNP-guided methylation of target RNAs [56].

Hence, box motifs are annotated based on both known pattern of conserved nucleotides and likely spacer distances, usually 12nt, between the box C/D and D’/C’ motifs. Only snoRNAs with boxes that could be annotated with high certainty are selected for the initial query set. The sequences are then grouped into gene families based on known orthology and sequence similarity.

### Homology search

In the next step all snoRNA families were mapped to all plant genomes. The list of all genomes with accession numbers is provided as Additional file 10. The snoStrip pipeline [33] was used to search each of the 24 plant genomes for homologs of each of the query families. In a nutshell, snoStrip is an automatic annotation pipeline that is developed specifically for comparative genomics of snoRNAs. It first uses both a blast search with relaxed parameters and infernal [58] to retrieve initial candidates.

The expected boxes and the anti-sense elements were annotated based on sequence alignments, and candidates were filtered for the presence of the boxes. The snoRNA fasta files along with coordinates of annotated snoRNAs are provided as Additional file 11. Then secondary structure features were validated. As part of the snoStrip pipeline RNAsubopt [59] is used for constraint folding. In the final step a family-wide alignment of all retained candidate sequences was calculated. The alignments produced by snoStrip are manually inspected. The respective alignments are provided as STOCKHOLM formatted files in Additional file 9.

Data were then aggregated to heatmaps showing the number of family members in each species. SnoRNA clusters were identified by proximities of genomic coordinates.

The history of gains and losses in each snoRNA family was reconstructed using a Dollo parsimony approach implemented in the ePoPe programm [47].

Since the nomenclature of plant snoRNAs only partially respects known or detectable sequence homology we used a unique internal family identifier throughout this study. These identifiers are re-translated to a consolidated family nomenclature that is based, in this order, on the nomenclature for *Arabidopsis*, *Oryza*, and *Chlamydomonas*. A complete table of family names and their species-specific synonyms is provided as Additional file 8.

## Endnote

^1^ https://en.wikipedia.org/wiki/Stockholm_format

## Additional files

Additional file 1 Relevant to box C/D snoRNAs heatmap. The.csv files contain the box C/D snoRNA families of the plant species that are represented in the heatmaps. (CSV 601 kb)

Additional file 2 Relevant to box H/ACA snoRNAs heatmap. The.csv files contain the box H/ACA snoRNA families of the plant species that are represented in the heatmaps. (CSV 211 kb)

Additional file 3 Alignments (in.aln format) representing co-evolution of conserved snoRNA-rRNA target interactions. (ZIP 109 kb)

Additional file 4 SnoRNA clusters. The folder includes figures (in.eps format) of all identified additional snoRNA clusters. (ZIP 4505 kb)

Additional file 5 Targets. Complete archive of the rRNA and snRNA targets is provided. These are.txt files, which include *RNAsnoop* and *Plexy* output. (ZIP 139 kb)

Additional file 6 ePoPE output details of box C/D snoRNAs. Detailed analysis of the predicted box C/D snoRNAs, lost genes, and lost families as outputted by ePoPE. (ODT 176 kb)

Additional file 7 ePoPE output details of box H/ACA snoRNAs. Detailed analysis of the predicted box H/ACA snoRNAs, lost genes, and lost families as outputted by ePoPE. (ODT 16.9 kb)

Additional file 8 Nomenclature. A complete table (.csv-formatted) of family names and their species-specific synonyms is provided. (CSV 209 kb)

Additional file 9 Alignments. Complete archive of all snoRNA family alignments (in.stk stockholm format). (ZIP 194 kb)

Additional file 10 List of Genomes and Accession Numbers. The list of all genomes with the accession numbers are added here for all the plants including red algae (.csv format). (CSV 284 kb)

Additional file 11 snoRNAs with coordinates. Complete archive of all fasta files of annotated snoRNAs is provided. The header of each sequence follows convention of the *snoStrip* pipeline and includes genome coordinates, detailes about the successful query during homology search and annotation of the characteristic box motifs. (ZIP 355 kb)

## Abbreviations

ePoPE: Efficient prediction of paralog evolution
K-turn: Kink-turn
miRNA: Micro RNA
mRNA: Messenger RNA
NCBI: National Centre for Biotechnology information
rRNA: Ribosomal RNA
Rfam: RNA family database
snoRNA: Small nucleolar RNA
snRNA: Small nuclear RNA
scaRNA: Small Cajal body-specific RNA
snoRNP: small nucleolar ribonucleoprotein
tRNA: Transfer RNA
