# Supplementary material for: Phylogenetic distribution of plant snoRNA families
Source: BMC Genomics. 2016 Nov 24;17:969. doi: 10.1186/s12864-016-3301-2 (PMC5122169; doi:10.1186/s12864-016-3301-2)
Supplement: Supplementary file 1 [file 12864_2016_3301_MOESM6_ESM.odt]

pos:   0 label:   0 kids: 1,2 pInP:   0 par: -1 snoRNAs:   0 gain:   0 loss   0	 seenBW:   1 gainFam:   0	 lossFam:   0	 Eukaryota

pos:   1 label:   1 kids: -1 pInP:   1 par:   0 snoRNAs:   0 gain:   0 loss   0	 seenBW:   1 gainFam:   0	 lossFam:   0	 cme

pos:   2 label:   2 kids: 3,4 pInP:   2 par:   0 snoRNAs:  16 gain:  16 loss   0	 seenBW:  -1 gainFam:   2	 lossFam:   0	 Viridiplantae

pos:   3 label:   3 kids: -1 pInP:   3 par:   2 snoRNAs: 173 gain: 170 loss  13	 seenBW:  -1 gainFam:  71	 lossFam:   0	 cre

pos:   4 label:   4 kids: 5,6 pInP:   4 par:   2 snoRNAs:  39 gain:  23 loss   0	 seenBW:  -1 gainFam:   7	 lossFam:   0	 Embryophyta

pos:   5 label:   5 kids: 7,8 pInP:   5 par:   4 snoRNAs:  26 gain:   0 loss  13	 seenBW:  -1 gainFam:   0	 lossFam:   0	 Tracheophyta

pos:   6 label:   6 kids: -1 pInP:  44 par:   4 snoRNAs:  34 gain:   0 loss   5	 seenBW:  -1 gainFam:   0	 lossFam:   1	 ppa

pos:   7 label:   7 kids: -1 pInP:   6 par:   5 snoRNAs:   6 gain:   0 loss  20	 seenBW:  -1 gainFam:   0	 lossFam:   6	 smo

pos:   8 label:   8 kids: 10,9 pInP:   7 par:   5 snoRNAs:  45 gain:  19 loss   0	 seenBW:  -1 gainFam:   9	 lossFam:   0	 Spermatophyta

pos:   9 label:   9 kids: 11,12 pInP:   9 par:   8 snoRNAs:  74 gain:  31 loss   2	 seenBW:  -1 gainFam:  11	 lossFam:   0	 Magnoliophyta

pos:  10 label:  10 kids: -1 pInP:   8 par:   8 snoRNAs:  37 gain:   0 loss   8	 seenBW:  -1 gainFam:   0	 lossFam:   1	 pta

pos:  11 label:  11 kids: -1 pInP:  10 par:   9 snoRNAs:  44 gain:   2 loss  32	 seenBW:  -1 gainFam:   0	 lossFam:   5	 atr

pos:  12 label:  12 kids: 13,14 pInP:  11 par:   9 snoRNAs: 128 gain:  54 loss   0	 seenBW:  -1 gainFam:  23	 lossFam:   0	 Mesangiospermae

pos:  13 label:  13 kids: 15,16,17 pInP:  12 par:  12 snoRNAs: 112 gain:   9 loss  25	 seenBW:  -1 gainFam:   9	 lossFam:   1	 Pentapetalae

pos:  14 label:  14 kids: 18,19 pInP:  33 par:  12 snoRNAs: 143 gain:  16 loss   1	 seenBW:  -1 gainFam:   1	 lossFam:   0	 commelinids

pos:  15 label:  15 kids: 20,21,22 pInP:  13 par:  13 snoRNAs: 125 gain:  25 loss  12	 seenBW:  -1 gainFam:   6	 lossFam:   2	 rosids

pos:  16 label:  16 kids: -1 pInP:  27 par:  13 snoRNAs:  87 gain:  11 loss  36	 seenBW:  -1 gainFam:   0	 lossFam:  13	 bvu

pos:  17 label:  17 kids: 26,27 pInP:  28 par:  13 snoRNAs: 111 gain:  18 loss  19	 seenBW:  -1 gainFam:   0	 lossFam:  10	 lamiids

pos:  18 label:  18 kids: -1 pInP:  34 par:  14 snoRNAs:  88 gain:   2 loss  57	 seenBW:  -1 gainFam:   0	 lossFam:  11	 pda

pos:  19 label:  19 kids: 31,32 pInP:  35 par:  14 snoRNAs: 194 gain:  54 loss   3	 seenBW:  -1 gainFam:  12	 lossFam:   2	 Poaceae

pos:  20 label:  20 kids: -1 pInP:  14 par:  15 snoRNAs:  82 gain:  10 loss  53	 seenBW:  -1 gainFam:   0	 lossFam:  18	 vvi

pos:  21 label:  21 kids: 23,24,25 pInP:  15 par:  15 snoRNAs: 126 gain:   8 loss   7	 seenBW:  -1 gainFam:   0	 lossFam:   3	 malvids

pos:  22 label:  22 kids: 28,29,30 pInP:  21 par:  15 snoRNAs: 110 gain:   7 loss  22	 seenBW:  -1 gainFam:   0	 lossFam:   9	 fabids

pos:  23 label:  23 kids: -1 pInP:  16 par:  21 snoRNAs: 124 gain:  25 loss  27	 seenBW:  -1 gainFam:   0	 lossFam:  14	 gra

pos:  24 label:  24 kids: 37,38 pInP:  17 par:  21 snoRNAs: 133 gain:  11 loss   4	 seenBW:  -1 gainFam:  14	 lossFam:   2	 Brassicaceae

pos:  25 label:  25 kids: -1 pInP:  20 par:  21 snoRNAs:  64 gain:   1 loss  63	 seenBW:  -1 gainFam:   0	 lossFam:  16	 csi

pos:  26 label:  26 kids: -1 pInP:  29 par:  17 snoRNAs: 127 gain:  44 loss  28	 seenBW:  -1 gainFam:   0	 lossFam:  16	 dpu

pos:  27 label:  27 kids: 40,39 pInP:  30 par:  17 snoRNAs: 113 gain:  15 loss  13	 seenBW:  -1 gainFam:   0	 lossFam:   6	 Solanum

pos:  28 label:  28 kids: -1 pInP:  22 par:  22 snoRNAs:  85 gain:   1 loss  26	 seenBW:  -1 gainFam:   0	 lossFam:   8	 rco

pos:  29 label:  29 kids: 41,42 pInP:  23 par:  22 snoRNAs: 106 gain:  12 loss  16	 seenBW:  -1 gainFam:   0	 lossFam:   6	 Papilionoideae

pos:  30 label:  30 kids: -1 pInP:  26 par:  22 snoRNAs:  90 gain:   8 loss  28	 seenBW:  -1 gainFam:   0	 lossFam:   5	 ppe

pos:  31 label:  31 kids: 33,34 pInP:  36 par:  19 snoRNAs: 212 gain:  18 loss   0	 seenBW:  -1 gainFam:   5	 lossFam:   0	 BOP_Clade

pos:  32 label:  32 kids: 35,36 pInP:  41 par:  19 snoRNAs: 156 gain:   3 loss  41	 seenBW:  -1 gainFam:   0	 lossFam:   8	 Andropogoneae

pos:  33 label:  33 kids: -1 pInP:  37 par:  31 snoRNAs: 126 gain:   7 loss  93	 seenBW:  -1 gainFam:  25	 lossFam:   5	 osa

pos:  34 label:  34 kids: 43,44 pInP:  38 par:  31 snoRNAs: 387 gain: 177 loss   2	 seenBW:  -1 gainFam:   0	 lossFam:   2	 Triticeae

pos:  35 label:  35 kids: -1 pInP:  42 par:  32 snoRNAs: 194 gain:  56 loss  18	 seenBW:  -1 gainFam:   0	 lossFam:  10	 zma

pos:  36 label:  36 kids: -1 pInP:  43 par:  32 snoRNAs: 123 gain:   2 loss  35	 seenBW:  -1 gainFam:   0	 lossFam:   0	 sbi

pos:  37 label:  37 kids: -1 pInP:  18 par:  24 snoRNAs: 289 gain: 161 loss   5	 seenBW:  -1 gainFam:   0	 lossFam:   1	 bra

pos:  38 label:  38 kids: -1 pInP:  19 par:  24 snoRNAs: 127 gain:  29 loss  35	 seenBW:  -1 gainFam:  27	 lossFam:   3	 ath

pos:  39 label:  39 kids: -1 pInP:  32 par:  27 snoRNAs: 115 gain:  19 loss  17	 seenBW:  -1 gainFam:   0	 lossFam:   2	 stu

pos:  40 label:  40 kids: -1 pInP:  31 par:  27 snoRNAs: 109 gain:  11 loss  15	 seenBW:  -1 gainFam:   0	 lossFam:   0	 sly

pos:  41 label:  41 kids: -1 pInP:  24 par:  29 snoRNAs:  90 gain:   7 loss  23	 seenBW:  -1 gainFam:   0	 lossFam:   5	 lja

pos:  42 label:  42 kids: -1 pInP:  25 par:  29 snoRNAs: 138 gain:  50 loss  18	 seenBW:  -1 gainFam:   0	 lossFam:   3	 mtr

pos:  43 label:  43 kids: -1 pInP:  39 par:  34 snoRNAs: 1213 gain: 829 loss   3	 seenBW:  -1 gainFam:   0	 lossFam:   1	 tae

pos:  44 label:  44 kids: -1 pInP:  40 par:  34 snoRNAs: 384 gain:  23 loss  26	 seenBW:  -1 gainFam:   0	 lossFam:   0	 hvu
