# Supplementary material for: Phylogenetic distribution of plant snoRNA families
Source: BMC Genomics. 2016 Nov 24;17:969. doi: 10.1186/s12864-016-3301-2 (PMC5122169; doi:10.1186/s12864-016-3301-2)
Supplement: Supplementary file 2 [file 12864_2016_3301_MOESM7_ESM.odt]

pos:   0 lab:   0 kids: 1,2 pInP:   0 par: -1 snoRNAs:  13 gain:  13 loss   0	 seenBW:  -1 gainFam:   1	 lossFam:   0	 Eukaryota

pos:   1 lab:   1 kids: -1 pInP:   1 par:   0 snoRNAs:   1 gain:   0 loss  12	 seenBW:  -1 gainFam:   0	 lossFam:   0	 cme

pos:   2 lab:   2 kids: 3,4 pInP:   2 par:   0 snoRNAs:  13 gain:   0 loss   0	 seenBW:  -1 gainFam:   0	 lossFam:   0	 Viridiplantae

pos:   3 lab:   3 kids: -1 pInP:   3 par:   2 snoRNAs: 110 gain: 110 loss  13	 seenBW:  -1 gainFam:  41	 lossFam:   1	 cre

pos:   4 lab:   4 kids: 5,6 pInP:   4 par:   2 snoRNAs:  21 gain:   8 loss   0	 seenBW:  -1 gainFam:   3	 lossFam:   0	 Embryophyta

pos:   5 lab:   5 kids: 7,8 pInP:   5 par:   4 snoRNAs:  21 gain:   0 loss   0	 seenBW:  -1 gainFam:   0	 lossFam:   0	 Tracheophyta

pos:   6 lab:   6 kids: -1 pInP:  44 par:   4 snoRNAs:  17 gain:   0 loss   4	 seenBW:  -1 gainFam:   0	 lossFam:   0	 ppa

pos:   7 lab:   7 kids: -1 pInP:   6 par:   5 snoRNAs:  14 gain:   0 loss   7	 seenBW:  -1 gainFam:   0	 lossFam:   1	 smo

pos:   8 lab:   8 kids: 10,9 pInP:   7 par:   5 snoRNAs:  28 gain:   7 loss   0	 seenBW:  -1 gainFam:   2	 lossFam:   0	 Spermatophyta

pos:   9 lab:   9 kids: 11,12 pInP:   9 par:   8 snoRNAs:  42 gain:  16 loss   2	 seenBW:  -1 gainFam:   3	 lossFam:   0	 Magnoliophyta

pos:  10 lab:  10 kids: -1 pInP:   8 par:   8 snoRNAs:  40 gain:  15 loss   3	 seenBW:  -1 gainFam:   0	 lossFam:   1	 pta

pos:  11 lab:  11 kids: -1 pInP:  10 par:   9 snoRNAs:  48 gain:  22 loss  16	 seenBW:  -1 gainFam:   0	 lossFam:   1	 atr

pos:  12 lab:  12 kids: 13,14 pInP:  11 par:   9 snoRNAs:  34 gain:   6 loss  14	 seenBW:  -1 gainFam:   5	 lossFam:   0	 Mesangiospermae

pos:  13 lab:  13 kids: 15,16,17 pInP:  12 par:  12 snoRNAs:  38 gain:   6 loss   2	 seenBW:  -1 gainFam:   2	 lossFam:   0	 Pentapetalae

pos:  14 lab:  14 kids: 18,19 pInP:  33 par:  12 snoRNAs:  41 gain:  11 loss   4	 seenBW:  -1 gainFam:   0	 lossFam:   1	 commelinids

pos:  15 lab:  15 kids: 20,21,22 pInP:  13 par:  13 snoRNAs:  41 gain:   9 loss   6	 seenBW:  -1 gainFam:   4	 lossFam:   0	 rosids

pos:  16 lab:  16 kids: -1 pInP:  27 par:  13 snoRNAs:  27 gain:   0 loss  11	 seenBW:  -1 gainFam:   0	 lossFam:   4	 bvu

pos:  17 lab:  17 kids: 26,27 pInP:  28 par:  13 snoRNAs:  31 gain:   2 loss   9	 seenBW:  -1 gainFam:   0	 lossFam:   3	 lamiids

pos:  18 lab:  18 kids: -1 pInP:  34 par:  14 snoRNAs:  27 gain:   1 loss  15	 seenBW:  -1 gainFam:   0	 lossFam:   3	 pda

pos:  19 lab:  19 kids: 31,32 pInP:  35 par:  14 snoRNAs:  43 gain:   5 loss   3	 seenBW:  -1 gainFam:   0	 lossFam:   0	 Poaceae

pos:  20 lab:  20 kids: -1 pInP:  14 par:  15 snoRNAs:  40 gain:  13 loss  14	 seenBW:  -1 gainFam:   0	 lossFam:   6	 vvi

pos:  21 lab:  21 kids: 23,24,25 pInP:  15 par:  15 snoRNAs:  40 gain:   1 loss   2	 seenBW:  -1 gainFam:   3	 lossFam:   0	 malvids

pos:  22 lab:  22 kids: 28,29,30 pInP:  21 par:  15 snoRNAs:  35 gain:   1 loss   7	 seenBW:  -1 gainFam:   0	 lossFam:   3	 fabids

pos:  23 lab:  23 kids: -1 pInP:  16 par:  21 snoRNAs:  51 gain:  16 loss   5	 seenBW:  -1 gainFam:   0	 lossFam:   4	 gra

pos:  24 lab:  24 kids: 37,38 pInP:  17 par:  21 snoRNAs:  40 gain:   1 loss   1	 seenBW:  -1 gainFam:   9	 lossFam:   0	 Brassicaceae

pos:  25 lab:  25 kids: -1 pInP:  20 par:  21 snoRNAs:  28 gain:   7 loss  19	 seenBW:  -1 gainFam:   0	 lossFam:   5	 csi

pos:  26 lab:  26 kids: -1 pInP:  29 par:  17 snoRNAs:  37 gain:  19 loss  13	 seenBW:  -1 gainFam:   0	 lossFam:   5	 dpu

pos:  27 lab:  27 kids: 40,39 pInP:  30 par:  17 snoRNAs:  34 gain:   6 loss   3	 seenBW:  -1 gainFam:   0	 lossFam:   2	 Solanum

pos:  28 lab:  28 kids: -1 pInP:  22 par:  22 snoRNAs:  27 gain:   0 loss   8	 seenBW:  -1 gainFam:   0	 lossFam:   1	 rco

pos:  29 lab:  29 kids: 41,42 pInP:  23 par:  22 snoRNAs:  35 gain:   3 loss   3	 seenBW:  -1 gainFam:   0	 lossFam:   1	 Papilionoideae

pos:  30 lab:  30 kids: -1 pInP:  26 par:  22 snoRNAs:  29 gain:   0 loss   6	 seenBW:  -1 gainFam:   0	 lossFam:   1	 ppe

pos:  31 lab:  31 kids: 33,34 pInP:  36 par:  19 snoRNAs:  44 gain:   2 loss   1	 seenBW:  -1 gainFam:   0	 lossFam:   0	 BOP_Clade

pos:  32 lab:  32 kids: 35,36 pInP:  41 par:  19 snoRNAs:  30 gain:   0 loss  13	 seenBW:  -1 gainFam:   0	 lossFam:   2	 Andropogoneae

pos:  33 lab:  33 kids: -1 pInP:  37 par:  31 snoRNAs:  27 gain:   0 loss  17	 seenBW:  -1 gainFam:   0	 lossFam:   2	 osa

pos:  34 lab:  34 kids: 43,44 pInP:  38 par:  31 snoRNAs:  78 gain:  34 loss   0	 seenBW:  -1 gainFam:   0	 lossFam:   0	 Triticeae

pos:  35 lab:  35 kids: -1 pInP:  42 par:  32 snoRNAs:  26 gain:   3 loss   7	 seenBW:  -1 gainFam:   0	 lossFam:   1	 zma

pos:  36 lab:  36 kids: -1 pInP:  43 par:  32 snoRNAs:  28 gain:   1 loss   3	 seenBW:  -1 gainFam:   0	 lossFam:   0	 sbi

pos:  37 lab:  37 kids: -1 pInP:  18 par:  24 snoRNAs: 101 gain:  64 loss   3	 seenBW:  -1 gainFam:   0	 lossFam:   4	 bra

pos:  38 lab:  38 kids: -1 pInP:  19 par:  24 snoRNAs:  47 gain:  17 loss  10	 seenBW:  -1 gainFam:   1	 lossFam:   0	 ath

pos:  39 lab:  39 kids: -1 pInP:  32 par:  27 snoRNAs:  28 gain:   0 loss   6	 seenBW:  -1 gainFam:   0	 lossFam:   0	 stu

pos:  40 lab:  40 kids: -1 pInP:  31 par:  27 snoRNAs:  43 gain:  13 loss   4	 seenBW:  -1 gainFam:   0	 lossFam:   1	 sly

pos:  41 lab:  41 kids: -1 pInP:  24 par:  29 snoRNAs:  33 gain:   9 loss  11	 seenBW:  -1 gainFam:   0	 lossFam:   1	 lja

pos:  42 lab:  42 kids: -1 pInP:  25 par:  29 snoRNAs:  29 gain:   4 loss  10	 seenBW:  -1 gainFam:   0	 lossFam:   3	 mtr

pos:  43 lab:  43 kids: -1 pInP:  39 par:  34 snoRNAs: 227 gain: 150 loss   1	 seenBW:  -1 gainFam:   0	 lossFam:   0	 tae

pos:  44 lab:  44 kids: -1 pInP:  40 par:  34 snoRNAs:  82 gain:   7 loss   3	 seenBW:  -1 gainFam:   0	 lossFam:   0	 hvu
